# Genome-Wide Identification of CircRNAs of Infective Larvae and Adult Worms of Parasitic Nematode, *Haemonchus contortus*

**DOI:** 10.3389/fcimb.2021.764089

**Published:** 2021-11-22

**Authors:** Caixian Zhou, Yao Zhang, Simin Wu, Zhiheng Wang, Waresi Tuersong, Chunqun Wang, Feng Liu, Min Hu

**Affiliations:** State Key Laboratory of Agricultural Microbiology, College of Veterinary Medicine, Huazhong Agricultural University, Wuhan, China

**Keywords:** *Haemonchus contortus*, circular RNA, developmental stage/gender, signal transduction, miRNA sponge

## Abstract

CircRNAs, a novel class of ncRNA family, are endogenous transcriptional products involved in various biological and physiological processes in plants and animals. However, almost no information is available for circRNAs of parasitic helminths. In the present study, the circRNAs repertoire was comprehensively explored in *Haemonchus contortus*, a blood-sucking parasitic nematode of ruminants. In total, 20073 circRNAs were identified and annotated from three key developmental stages/genders of *H. contortus* including the free-living infective third-stage larvae (L3, 18883), parasitic adult female (Af, 3491), and male worms (Am, 2550) *via* deep-sequencing technology and bioinformatic analysis. Among these identified circRNAs, 71% were derived from exonic regions of protein-coding genes. The number of circRNAs transcribed from the X chromosome (4704) was higher than that from Chromosome I-V (3143, 3273, 3041, 3030, 2882). The amount of highly expressed circRNAs in third-stage larvae was significantly more abundant than that in adult stage. 15948 and 16847 circRNAs were differentially expressed between Af and L3s and between Am and L3, respectively. Among them, 13409 circRNAs existed in both comparisons. Furthermore, 1119 circRNAs were differentially expressed between Af_and_Am. GO enrichment analysis indicated that source genes of circRNAs differentially expressed between Am and L3 as well as between Af and L3 were significantly enriched in many biological processes, primarily including signaling, signal transduction and cell communication terms. KEGG analysis revealed that parental genes of differentially expressed circRNAs were mainly related to metabolism (pyruvate metabolism, glycerophospholipid metabolism, and carbon metabolism), MAPK signaling pathway, and phosphatidylinositol signaling system. Moreover, many circRNAs contained one or more miRNA potential binding sites, suggesting that they could regulate gene expression at the post-transcriptional level. Furthermore, the correctness of head-to-tail back splicing site and alternative circularization events were verified by Sanger sequencing using both divergent and convergent primers. Finally, the reliability of RNA-Seq data and the resistance of circRNAs to RNase R digestion were confirmed by quantitative RT-PCR. Taken together, our findings provide a foundation for elucidating the regulatory mechanisms of circRNAs in *H. contortus*, which will advance the understanding of circRNAs in parasitic nematodes.

## Introduction

Circular RNAs (circRNAs), a novel class of endogenous non-coding RNAs with a covalently closed loop structure without 3′ end poly-adenylated tail and the 5′ end cap structure, have been widely discovered in the eukaryotic transcriptome. Although circRNAs with circular structure in eukaryotic cells were observed by electron microscopy more than four decades ago (Hsu and Coca-Prados, 1979), and have been reported sporadically thereafter (Grabowski et al., 1981; Capel et al., 1993), circRNAs were deemed to be functionless side-products of transcription or artifacts of aberrant RNA splicing. Owing to dramatic advances of high-throughput sequencing technology and specialized bioinformatic analysis pipelines, many circRNAs have been frequently recognized in diverse species, such as mammals (Guo et al., 2014), *Drosophila* (Westholm et al., 2014), *Caenorhabditis elegans* (Memczak et al., 2013), plants (Zhang et al., 2020) and Archaea (Danan et al., 2012). Characteristics analysis revealed that circRNAs could be generated from exonic, intronic, intergenic regions, and exonic-intronic regions through non-canonical splicing event called back-splicing (Li et al., 2018a; Kristensen et al., 2019), and the expression patterns were often cell, tissue, and developmental stage-specific (Salzman et al., 2013; Liang et al., 2017; Nicolet et al., 2018). The research on circRNAs has become one of the amazing hotspots in life science and the knowledge about circRNAs has been renovated uninterruptedly and fleetly.

More recently, the biological roles of circRNAs were continuously illuminated. Firstly, one of the prominent roles is that abundant circRNAs located in the cytoplasm and harbored miRNA binding sites can serve as miRNA sponges or decoys to regulate gene expression. For instance, in the mouse brain, ciRS-7/CDR1as was demonstrated to be a miR-7 sponge and to modulate miR-7’s biological activities and functions (Hansen et al., 2013). Secondly, some circRNAs can also act as protein sponges or decoys to indirectly regulate protein’s functions. For example, in mouse embryo fibroblast and cardiac fibroblast, circ-Foxo3 retarded cell cycle progression through interacting with p21 and CDK2 (Du et al., 2016). Thirdly, a novel subclass of circRNAs, ElciRNAs, have been demonstrated to promote the transcription of their parent genes by interacting with U1 snRNP and RNA Polymerase II in the promoter region (Li et al., 2015). Except for regulating gene transcription and protein expression, some circRNAs can be directly translated into detectable proteins. For example, in murine and human myoblast, circ-ZNF609 could be translated into a protein to control myoblast proliferation (Legnini et al., 2017). However, for most circRNAs, their functions remain unknown.

In contrast to vertebrate and plants, to date, the research on circRNAs in nematode is still limited. Only in the free-living nematode *C. elegans*, circRNAs have been identified from different developmental stages including sperm, oocytes, one/two-cell embryos (Memczak et al., 2013), the fourth-stage larvae (L4) (Cortes-Lopez et al., 2018), and adults (Ivanov et al., 2015). However, up to date, there is no information available for circRNAs of parasitic nematodes including the blood-sucking nematode *Haemonchus contortus*, which infects small ruminants and causes significant economic losses to the livestock industry around the world. This parasite has a direct and rapid life cycle (Laing et al., 2013; Schwarz et al., 2013). The adult worms live in the mucosa of host abomasum and females produce eggs that are excreted from host feces. The eggs hatch and develop to the infective third-stage larvae (L3) through first- and second-stage larvae in around seven days. The L3s are transmitted orally from contaminated herbage to the grazing host, exsheath, and develop to dioecious adults through L4 in two to three weeks. L4s and adult worms feed on blood from capillaries in the ruminant abomasum mucosa.

In order to study whether circRNAs play a key role in the development of gastrointestinal parasitic nematodes, it is necessary to analyze the expression and differences of circRNAs in different developmental stages/sexes. In the current study, circRNAs were sequenced from L3s, female and male worms of *H. contortus*. Their genomic feature, length distribution, and the number of exons coding for circRNAs were analyzed. Bioinformatic functional analyses of circRNAs were also conducted. In addition, Sanger sequencing was employed to validate the head-to-tail back-splicing site, and qRT-PCR was used to verify the differentially expressed circRNAs and the resistance of circRNAs to RNase R digestion. This is the first identification of circRNAs in parasitic nematodes, the data provide a basis for further studying the functions of circRNAs in *H. contortus* and other related nematode species.

## Materials and Methods

### Parasite Sample Collection

*Haemonchus contortus* L3s and adult worms were collected as described previously (He et al., 2019). Briefly, experimental goats (3 months of age) purchased from Hubei Academy of Agricultural Sciences were confirmed helminth-free by fecal examination and maintained under a parasite-free condition. The goats were orally inoculated with 8000 infective L3 of *H. contortus* (Haecon-5). Four weeks post-infection, fecal samples were harvested and incubated at 27°C for 1 week, then iL3s were collected by the Baermann funnel method. Adult female and male worms were collected from the abomasum after euthanasia and necropsy of infected goats according to their distinctive appearances. All samples were washed thoroughly in sterile phosphate-buffered saline and then transferred to liquid nitrogen for storage until use.

### Extracting RNA, Strand-Specific Library Preparation, and Sequencing

Total RNA samples were extracted, respectively, from three independent experimental replicates of L3s (40,000 larvae per replicate), male adults (Am), and female adults (Af) (30 worms per replicate) of *H. contortus* using *TranZol* (Sigmen, China) with the manufacturer’s instructions. The total RNA yields and quality were evaluated on the Agilent 2100 Bioanalyzer (Agilent, USA). Then, approximately 5 μg of total RNA from each sample was treated to remove ribosomal RNA (rRNA) using Ribo-zero™ rRNA Removal Kit (Illumina, USA) following the manufacturer’s procedure, and further incubated with RNase R (Epicentre, USA) to digest linear RNAs. The remaining RNAs were used as templates for generating circRNA-seq libraries. Finally, all eligible libraries were sequenced using the Illumina HiSeq X Ten platform to generate 100 bp pair-end reads at BGI Technology Co. Ltd (Shenzhen, China). The samples were named as Af_1, Af_2, Af_3; Am_1, Am_2, Am_3; L3_1, L3_2, L3_3, respectively.

### Identification of Differently Expressed circRNAs

Firstly, all the raw data were removed with low-quality reads, unknown bases and adaptors, and then FastQC (http://www.bioinformatics.babraham.ac.uk/projects/fastqc/) was used to confirm the quality of reads. The Q20, Q30, and GC contents were also simultaneously calculated. Subsequently, Bowtie2 (Langmead et al., 2009) and BWA (Li and Durbin, 2009) were employed to map the clean reads to the publicly available *H. contortus* reference genomes, which was downloaded from WormBase Parasite website (https://parasite.wormbase.org/Haemonchus_contortus_prjeb506/). Lastly, find_circ (Memczak et al., 2013) and CIRI2 (Gao et al., 2018) tools with default parameters were performed to identify circRNAs. Only the candidate circRNAs were identified by both algorithm methods in all replicates for further calculation of expression level.

The expression level of circRNAs from each sample was quantified based on the read counts, normalized using transcripts per million (TPM) as previously described (Zhou et al., 2010) and calculated as the normalized expression level = (read count*1,000,000)/the sum of circRNAs read count. The differentially expressed circRNAs were identified by pairwise comparison between L3s, adult female and male worms using DESeq2 algorithm (Love et al., 2014), and the cut-off criterion was set as |log_2_^FoldChange^| ≥ 1 and padj < 0.05. Venn, Heat map clustering, circos, and volcano diagrams were drawn using correspondence R packages.

### Bioinformatics Functional Analysis

Based on the Gene Ontology (GO) (Young et al., 2010) and Kyoto Encyclopedia of Genes and Genomes (KEGG) (Kanehisa et al., 2017) databases, the potential functional analysis of all the parental genes of differentially expressed circRNA was carried out. GO annotation was performed by BLAST (E-value < 10^-5^) using C. elegans orthologs, which was then assigned to terms using clusterProfiler R packages (Yu et al., 2012). The padj<0.05 denotes the threshold representing the significantly enriched GO terms. All enriched GO terms that possessed a padj < 0.05 were displayed. The KOBAS (http://kobas.cbi.pku.edu.cn/) was used to test the statistical enrichment of the circRNA-host genes in the KEGG pathways. The top 20 enriched pathways were selected based on the p-value and rich factor.

### The Construction of circRNA-miRNA Network

To determine whether circRNAs in *H. contortus* could function as miRNA sponges to regulate their target mRNAs, 194 mature miRNA sequences of *H. contortus* were downloaded from miRbase 22 (https://mirbase.org/ftp.shtml). The miRNA targeting sites for circRNAs were identified by miRanda algorithm (Enright et al., 2003) with threshold parameters as follows: max free energy values <− 20 and a score of 150 or higher. Based on the predicted results, network of circRNA–miRNA was then constructed and visualized using Cytoscape 3.7.1 software (Shannon et al., 2003).

### Validation of Back-Splicing Site of circRNAs

The total genomic DNA (gDNA) was extracted from *H. contortus* using the Blood/Cell/Tissue Genomic DNA Extraction Kit (Tiangen, China) with the manufacturer’s instructions. The total RNA was extracted from L3s, adult female or male worms of *H. contortus*, respectively and then cDNA was reverse transcribed using HiScript II Q RT SuperMix (Vazyme, China) following the manufacturer’s protocol.

CircRNAs were validated by PCR as previously described (Memczak et al., 2013). To do it, divergent and convergent primers were designed, respectively. Linear transcripts were detected by convergent primers, and the candidate circular templates were detected using divergent primers. If the band with expected size was amplified with convergent primers from both cDNA and gDNA templates, but only amplified with divergent primers from cDNA template, it would suggest the presence of the back-splicing junction. For each PCR amplification, cDNA or gDNA was used with 2×Taq Master Mix (Vazyme, China), and 40 cycles of PCR cycling condition were performed. PCR products were examined by 1% agarose gel electrophoresis, and those with expected bands and amplified with divergent primers only from cDNA templates were sent to TSING Ke Biotech Co., Ltd. (Wuhan, China) for Sanger sequencing. Both specific divergent and convergent primers were synthesized by TSING Ke Biotech Co., Ltd. (Wuhan, China), and the primer information was listed in **Additional File 1**: **Table S1**.

### Quantification of circRNAs With Quantitative Real-Time PCR

To test accuracy of high-throughput sequencing, differentially expressed circRNAs with the junction site verified were selected in each comparison group to check by qRT-PCR assay. In addition, for confirmation of the resistance of circRNAs to the exoribonuclease RNase R, the qRT-PCR experiment was also performed using RNA samples with or without RNase R treatment. The total RNA was incubated with 4U/μg of RNase R (RNR07250, Epicentre) for 15 min at 37°C, 10 min at 70°C, and mock treatment was carried out in the same conditions without RNase R treatment. The qRT-PCR experiment was performed using 2× SYBR Green Master Mix (Takara, China), and the expression levels of circRNAs was determined with divergent primers using the ViiA™ 7 Real-Time PCR System (Applied Biosystems, USA). 2^-△△Ct^ method was used to calculate the relative expression level of each target circRNA and the *β-tubulin* gene was an internal control. The qRT-PCR experiment was carried out with the following reaction system: 10 μL of the 2× SYBR Green, 0.4 μL dye, 8 μL of the cDNA template and RNase-free ddH_2_O, 0.8 μL of the forward and reverse primers, respectively. The divergent primers were synthesized by TSING Ke Biotech Co., Ltd. (Wuhan, China), and the primer information was listed in **Additional File 1**: **Table S2**.

### Statistical Analysis

These works were created by GraphPad Prism 9.0 (GraphPad Company, USA). Student’s t-test was performed to compare the expression levels of target circRNAs in L3s, adult female and male worms and the analysis of the results was with the mean ± SEM. The results were statistically significant at *P<0.05, **P<0.01, ***P < 0.001, ****P < 0.0001; ns, no significant.

## Results

### General Properties of *H. contortus* circRNAs

**Figure 1:**
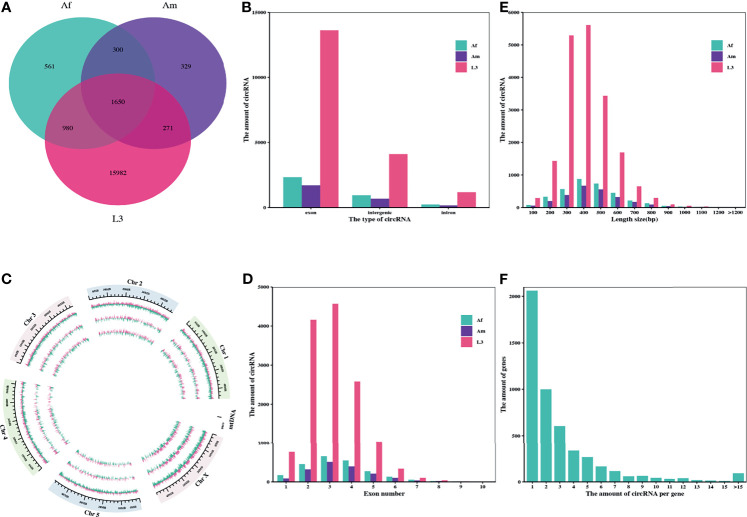
Characteristics of the identified circRNAs in *H. contortus*. **(A)** Venn diagram presenting the amount of circRNAs in third-stage larvae (L3), adult female worms (Af), and adult male worms (Am). **(B)** The numbers of circRNAs derived from exonic, intronic, and intergenic regions in L3, Af, and Am, respectively. **(C)** Circos plot showing the distribution of circRNAs on six *H*. *contoruts* chromosomes. Light pink bar represented circRNAs from plus strand; pale green bar from negative strand. Each track from the periphery to the core represents: chromosomes; L3; Af; Am. **(D)** Distribution of amount of circRNAs derived from different number of exons. **(E)** Length range distribution of the identified circRNAs. **(F)** The number of circRNAs per gene.

In order to identify *H. contortus* circRNAs, nine libraries were constructed and sequenced from L3s, adult female (Af) and male worms (Am) of this parasite. Clean data were used in subsequent analysis after wiping off the adaptor and low-quality reads. Among the nine libraries, the proportion of clean reads mapped to the *H. contortus* genome ranged from 61.07% to 74.73% (**Additional File 1**: **Table S3**). A total of 20073 candidate circRNAs with more than two back-spliced junction reads were identified and annotated from L3s, Af and Am worms using find_circ and CIRI2 computational pipelines (**Additional File 1**: **Table S4**). Among these circRNAs, 3491, 2550, and 18883 were detected in Af, Am, and L3 stages, respectively, and only 1650 were prevailed in all three stages/sexes, suggesting that most circRNAs in *H. contortus* were stage-specific (**Figure 1A**).

The subsequent analysis on genomic origin of circRNAs revealed that 14314 circRNAs (71.31%) were generated from exonic regions and 4476 circRNAs (22.30%) were generated from intergenic regions, but only 1283 circRNAs (6.39%) were stemmed from intronic regions (**Figure 1B**). In addition, the distribution of genome region coding for circRNAs on each chromosome was further examined using circlize R package. The mapping results showed that genome regions coding for these circRNAs were widely and unevenly distributed across the whole *H. contortus* chromosomes and were more abundant on the chromosome X than on other chromosomes. There is no correlation between the distribution of circRNAs and chromosome length (**Figure 1C**).

Next, the amount of exon-derived circRNAs were counted and showed that most of them typically encompass less than five exons. More than 94.18% of circRNAs were formed by multiple exons (up to 10 exons) (**Figure 1D**). Considering the length distribution, most circRNAs (83.48%) ranged from 200 bp to 600 bp (**Figure 1E**) and the average length of circRNAs was 354 bp, while the maximum length was 1875 bp. Moreover, the analysis on alternative circularization suggested that most genes yielded one or two circRNAs, some genes generated multiple distinguishing circularized products. 4937 genes encoded at least one circRNA, among them, 41.74% of genes only produced one circRNA, and 94.75% produced no more than 10 circRNAs (**Figure 1F**).

### Expression Analysis of circRNAs

**Figure 2:**
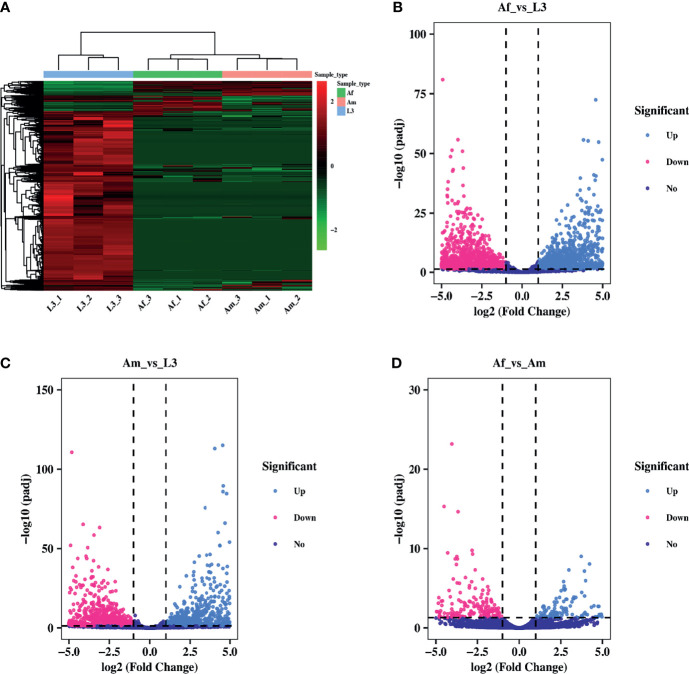
Differential expression pattern of circRNAs in *H. contortus*. **(A)** Hierarchical clustering analysis of differentially expressed circRNAs across L3, Af, and Am samples. The expression values were represented in different colors. Red strip represented high relative expression and green strip indicated low relative expression. **(B–D)** The volcano plot for differentially expressed circRNAs in Af_vs_L3, Am_vs_L3, Af_vs_Am comparisons. The volcano plot was constructed using log2 (Fold Change) and padj. light blue points: up-regulated circRNAs; red points: down-regulated circRNAs; purple points: no difference. The cut-off criterion was set as Fold Change ≥ 2 and padj< 0.05.

To analyze the expression pattern of circRNAs during *H. contortus* growth process, the expression level was quantified and differentially expressed circRNAs were screened by pairwise comparison between L3, adult female and male worms using DEGseq2. The hierarchical clustering presented the expression changes of circRNAs during three stages/sexes of *H. contoruts*. Overall, the abundance of most circRNAs decreased during development from L3 to adult stage (**Figure 2A**). 15948 circRNAs were identified differentially expressed between Af_vs_L3 group, shown in the volcano plot. Among them, 1707 were up-regulated and 14241 were down-regulated (**Figure 2B**). In the comparison between Am_vs_L3, 16847 circRNAs were identified as differentially expressed, including 1356 up-regulated and 15491 down-regulated (**Figure 2C**). Among the differentially expressed circRNAs, 13409 commonly existed in both Af_vs_L3 and Am_vs_L3 comparisons. In addition, comparison between Af_vs_Am revealed 1119 differentially expressed circRNAs. Among them, 637 were up-regulated and 482 were down-regulated (**Figure 2D**).

### Functional Annotation Analysis of Differentially Expressed circRNAs

**Figure 3:**
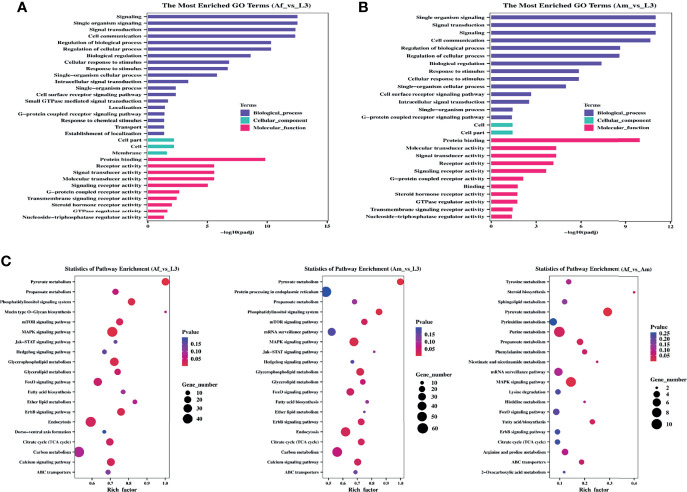
GO and KEGG enrichment analysis for differentially expressed circRNAs of *H. contortus*. **(A, B)** The most enriched GO terms in Af_vs_L3 and Am_vs_L3. Y-axis represented GO terms; X-axis represented the value of -log10 (padj). Different colors represented biological_process, cellular_component, and molecular_function, respectively. **(C)** The top 20 enriched KEGG pathways. Y-axis represents pathways; X-axis represents rich factor; The color and size of each bubble represent pvalue and the number of genes enriched in a pathway, respectively. Af_vs_L3, Am_vs_L3, and Af_vs_Am are represented from left to right.

Analysis of the function of parental genes could provide valuable clues about circRNA’s functions, thus, GO and KEGG annotation analyses were conducted. GO categories with padj less than 0.05 were assigned to the parental genes, and the significantly enriched genes of each GO term were statistically analyzed. In Af_vs_L3 group (**Figure 3A**), source genes of differentially expressed circRNAs were divided into thirty-two significant GO terms, nineteen of them were involved in the biological process category. The remarkably enriched GO terms include “signaling” (GO:0023052), “single organism signaling” (GO:0044700), and “signal transduction” (GO:0007165), “response to stimulus” (GO:0050896). Only three GO terms, “cell part” (GO:0044464), “cell” (GO:0005623), and “membrane” (GO:0016020), were annotated in cellular component subcategories. As for the molecular function, ten GO subcategories were enriched and mainly included “protein binding” (GO:0005515), “receptor activity” (GO:0004872), and “signal transducer activity” (GO:0004871). In Am_vs_L3 comparison group (**Figure 3B**), 14, two, and 11 terms were categorized into biological process, cellular component, and molecular function, respectively. The significant enrichment GO terms were like those of the Af_vs_L3 group and no significant enrichment GO terms were identified in the Af_vs_Am group.

Accordingly, the KEGG pathway analysis was further carried out to yield significantly enriched pathways. The top 20 enriched pathways are displayed in an enriched scatter diagram (**Figure 3C**). In Af_vs_L3 and Am_vs_L3 groups, the significantly represented pathways were pyruvate metabolism, MAPK signaling pathway, glycerophospholipid metabolism, and phosphatidylinositol signaling system, implying that source genes in *H. contortus* were mainly involved in signal processing and associated with the longevity of worm. However, the parent genes in Af_vs_Am comparison group were primarily related to metabolisms, such as pyruvate metabolism, propanoate metabolism, purine metabolism, fatty acid biosynthesis, and steroid biosynthesis.

### Construction of Complete Endogenous (ceRNA) Network

**Figure 4:**
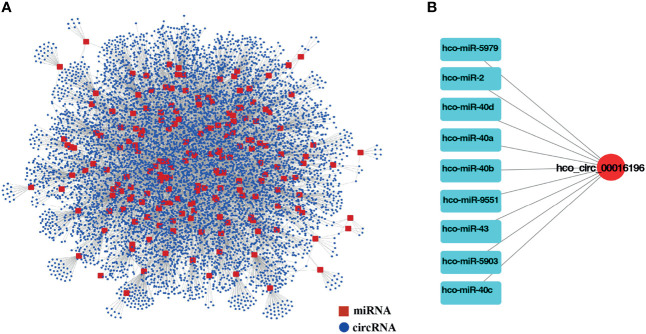
**(A)** The interaction network of differentially expressed circRNAs and miRNAs for *H*. *contortus.* Red squares represent miRNAs, and blue nodes represent circRNAs. **(B)** The circRNA, hco_circ_00016196, and nine miRNAs interaction network. Red nodes represent circRNAs, and light blue squares represent miRNAs.

Mounting researches reported that circRNAs might act as miRNAs sponges and likely modulate their activities (Yang et al., 2019; Tang et al., 2021; Xin et al., 2021). Meanwhile, miRNAs could be involved in multiple physiological processes during nematode development (Zhang et al., 2018; Mani et al., 2021). Information on the potential interaction between circRNA and miRNA will be helpful for understanding the function and regulatory mechanism of circRNA in *H. contortus*. However, the expression profiles of miRNAs were not available in the databases for different developmental stages of *H. contortus* (only 194 miRNAs available from L3 and mixed-sex adult worms), the potential miRNA targets can only be predicted based on sequence complementarity *via* miRanda algorithm. The predicted network consisted of 12015 interacting pairs and 8268 nodes, and the nodes included 8077 differentially expressed circRNAs and 191 miRNAs except for hco-miR-5892a, hco-miR-5892b, and hco-miR-5898-5p (**Figure 4A** and **Additional File 1**: **Table S5**). This indicated that a single miRNA could be targeted by different circRNAs, and that single circRNA could also sponge more than one miRNA. For example, hco-lin-4, hco-miR-1, and hco-miR-124 could be targeted by 36, 19, and 102 circRNAs, respectively. 55.89% of circRNAs contained more than one different miRNA-binding site, and hco_circ_00016196 harbored the greatest number (nine) of different miRNA binding sites (**Figure 4B**). The vast majority of circRNAs had one or two binding sites for one particular miRNA, only hco_circ_00015227 had 10 binding sites for one miRNA (hco-miR-307) (**Additional File 1**:**Table S6**).

### Validation of circRNAs by Sanger Sequencing

**Figure 5:**
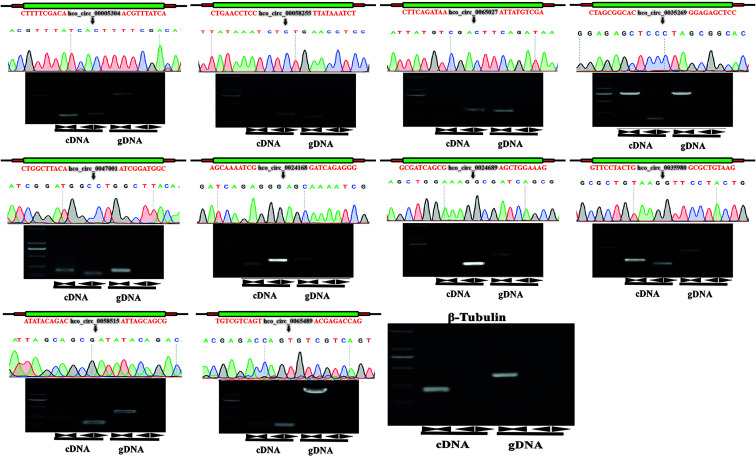
Experimental validation of back-spliced junction sites of candidate circRNAs. Linear RNAs were successfully amplified by convergent primers from both cDNA and gDNA. CircRNAs were successfully amplified by divergent primers from cDNA but failed to be amplified from gDNA. PCR products were detected by 1% agarose electrophoresis. The head-to-tail back-splicing site was further confirmed by Sanger sequencing. Black inverted triangles represented the back-spliced junction loci, and *β-tubulin* gene was used for the control group. Marker: 2000 bp ladder DNA marker.

For validating individual circRNA’s back-splicing junction site predicted from the analysis pipeline, divergent and convergent primers were designed for candidate circRNAs and used in PCR amplification with cDNA and gDNA as templates, respectively. From cDNA template, PCR products with expected sizes were amplified with both convergent primers (black opposing triangle pairs) and divergent primers (black back-to-back triangle pairs). However, from gDNA, PCR products with expected sizes were only exclusively amplified with convergent primers. The amplified products were then sequenced and the correctness of head-to-tail back splicing site was confirmed. In total, twenty-four circRNAs were verified. The original sequences of parental genes of 10 selected circRNAs and the sequencing chromatogram of PCR products amplified from cDNA or gDNA were shown (**Figure 5** and **Additional File 1**: **Figure S1**).

### Validation of circRNA Quantification and RNase R Digestion

**Figure 6:**
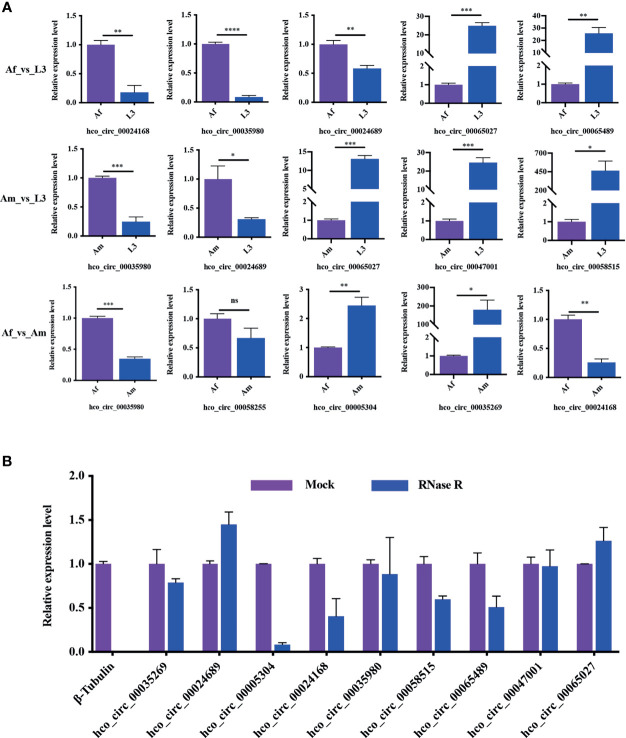
Reliability verification of RNA-Seq and the resistance of circRNAs to RNase R digestion. **(A)** The validation of differentially expressed circRNAs in all three comparisons. All experiments were conducted in three independent biological replicates and presented as means ± SEM. The expression levels among different groups were compared by Student’s t-test. *P < 0.05; **P < 0.01; ***P < 0.001, ****P < 0.0001. ns, no significant. **(B)** The results indicated that circRNAs were detected in both RNase R treated and control RNA samples, whereas the linear control *β-tubulin* gene wasn’t detected in RNase R treated sample. All experiments were conducted in two independent biological replicates and presented as means ± SEM.

To verify the reliability of high throughput sequencing data, differentially expressed circRNAs with the back-splicing junction sites validated were selected in each comparison group to assess the differential expression level by qRT-PCR (**Figure 6A**). In Af_vs_L3 group (three up-regulated and two down-regulated in adult female worms) and Am_vs_L3 group (two up-regulated and three down-regulated in adult male worms), the expression patterns of the selected circRNA detected by qRT-PCR were consistent with those from high-throughput sequencing results. In Af_vs_Am group (three up-regulated and two down-regulated circRNAs in adult female worms), qRT-PCR results also indicated a similar expression tendency with high-throughput sequencing results except for hco_circ_00058255 for which no difference was detected by qRT-PCR. In general, the correlation between qRT-PCR results and RNA-Seq is 0.85, suggesting that the high throughput sequencing results were dependable.

RNase R, a 3’ to 5’ exoribonuclease from the *E. coli* RNR superfamily, can digest almost all linear RNAs but not circRNAs. To assess the resistance of *H. contortus* circRNAs to RNase R digestion, total RNA was treated with RNase R and then used for cDNA synthesis followed by qRT-PCR detection. The transcription level of linear RNA control (*β-tubulin*) was significantly reduced after RNase R treatment, nevertheless, the transcription of circRNAs was only slightly decreased or increased (**Figure 6B**). This result confirmed that *H. contortus* circRNAs were endowed with a strong resistance to RNase R treatment.

### Alternative Circularization of circRNAs

**Figure 7:**
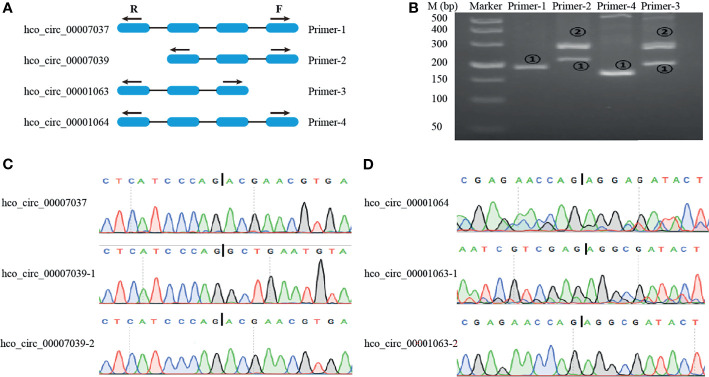
Visualization and validation of two alternative back-splicing circularization events. **(A)** A schematic diagram of the divergent primers designed for alternative splicing event. **(B)** Representative PCR products amplified with primers presented in **(A)** were examined by 3% agarose electrophoresis. M: 500 bp ladder DNA marker. **(C, D)** Sanger sequencing to validate the backsplice sites. Vertical Bar represents the backsplice junction site.

Different circRNAs can be generated from the same source gene consisting of different exons and/or introns. To do alternative splicing analysis, two-parent genes (HCON_00020120 and HCON_00002920) each generating two circRNA forms were selected. HCON_00020120 generated both hco_circ_00007037 and hco_circ_00007039, which had different acceptors but shared the same donor. In contrast, HCON_00002920 produced both co_circ_00001063 and hco_circ_00001064, which had different donors but shared the same acceptor. Divergent primers for these four circRNAs were designed as illustrated in **Figure 7A**. The PCR products were amplified using divergent primers from cDNA and then sequenced to validate the predicted back-splicing site. Primer-1 and primer-4 only amplified hco_circ_00007037 and hco_circ_00001064, respectively, but primer-2 and primer-3 each amplified two circRNAs (hco_circ_00007037 and hco_circ_00007039 by primer-2) and (hco_circ_00001063 and hco_circ_00001064 by primer-3), respectively (**Figure 7B**). The sizes of PCR products and sequences in the back-splicing site of each PCR product were confirmed by agarose gel electrophoresis and Sanger sequencing, respectively (**Figures 7B–D**). Primer sequence and length of PCR products were listed in **Additional File 1**: **Table S7**.

## Discussion

With the advances of sequencing technology, abundant resources of genomics and transcriptome have been accumulated for nematodes, while the research on the regulation of gene expression mediated by non-coding RNA in parasitic nematodes is still very scarce. High-throughput sequencing technology and bioinformatics methods make it possible to identify novel types of transcripts, even those often with low expression levels. Previous studies have identified the mRNA and miRNA expression profiles for key growth stages/sexes of *H. contortus* (Winter et al., 2012; Laing et al., 2013; Ma et al., 2018; Marks et al., 2019). Nevertheless, circRNAs, a recently discovered novel class of non-coding RNA from various animals and plants, have yet been studied in any parasitic nematodes including *H. contortus*. How circRNAs are transcribed in *H. contortus* and whether their expressions participate in the regulation of developmental processes of this parasite remain totally unknown. The present study represents the first one to perform genome-wide identification and potential function analysis of circRNAs in parasitic nematodes.

In the present study, from L3s, adult female and male worms of *H. contortus*, a total of 20073 circRNA were identified, the number of which is notably much higher than those from *C. elegans* (1166) (Cortes-Lopez et al., 2018) and *Plasmodium falciparum* (1381) (Broadbent et al., 2015). One of the reasons causing this was likely that our study performed an experimental strategy of ribo-depleted RNA-Seq coupled with RNase R enrichment. A previous study showed that from RNase R treated samples, indeed more candidate circRNAs were identified with approximately equal sequencing depth and prediction tools (Zeng et al., 2017). In our study, 2550, 3491, and 18883 circRNAs were detected in adult male, adult female, and L3 stages, respectively, and only 1650 circRNAs existed in all developmental stages/sexes, indicating that most circRNAs are stage-specific. However, what remains unclear is why the amount of circRNAs in L3 was significantly higher than those in the adult worms. We have checked out the analyses many times and ruled out the possibility of contamination. The reasons causing the significant difference in the number of circRNAs between L3 and adult worms hopefully can be discovered in the future when more circRNAs are identified from a wide range of parasitic nematode species.

Notoriously, based on the derivation of circRNAs in the genome, they can be divided into exonic, intronic, and intergenic ones (Kristensen et al., 2019). Among the identified 20073 circRNAs, the exon-derived were predominant (71.31%), consistent with the results from other species, such as *Arabidopsis thaliana* (Chen et al., 2017), *C. elegans* (Cortes-Lopez et al., 2018), zebrafish (Liu et al., 2019), and human (Xu et al., 2017). However, in wheat (Wang et al., 2016) and kiwifruit (Wang et al., 2016; Wang et al., 2017), intergenic circRNAs were in the majority, suggesting that the way that these genomes encode circRNAs occurs in a species-specific manner and the molecular basis of circRNA biogenesis is highly different among different species. In addition, a single gene locus could selectively generate multiple circRNAs through alternative back-splicing, and the events of alternative circularization phenomenon have been experimentally validated in human (Feng et al., 2019), fruit fly (Gao et al., 2016), and rice (Lu et al., 2015). Here, the alternative splicing events were analyzed and experimentally verified, indicating that such splicing mechanisms were conserved in parasitic nematodes.

CircRNAs were usually produced from protein-coding genes so that they may function in the same pathway as the source genes (Wilusz, 2017). The comparative analyses between adult worms and L3 revealed that significantly enriched GO terms for source genes were related to signaling, signal transduction, and response to stimulus, indicating that circRNAs could play roles in parasite’s response to environmental signals (e.g., temperature, humidity) during its transition from the free-living stage to the parasitic stage in order to adapt to the host abomasum environment. The KEGG pathway analysis identified several important pathways which are mainly involved in *H. contortus* growth, including MAPK signaling pathway, phosphatidylinositol signaling system, FoxO signaling pathway. In addition, interestingly, an identified exonic circRNA (hco_circ_00047001) was generated from the serine/threonine-specific protein kinase encoding gene, *akt*, which was known to associate with larval development (Di et al., 2020), suggesting that this circRNA may also play a role in larval development. Furthermore, hco_circ_00006206 was transcribed from HCON_00017820 (*Hc-aap-1*, Li et al., 2014), and four circRNAs (hco_circ_00012209, hco_circ_00012210, hco_circ_00012216, and hco_circ_00012219) derived from HCON_00034230 (*Hc-age-1*, Li et al., 2014). *Hc-aap-1* and *Hc-age-1* are important genes in the insulin-like signaling pathway of *H. contortus*, which play significant roles in the development of this parasite, especially during its transitional change from the environment to the host abomasum (Li et al., 2014; Li et al., 2016; Ma et al., 2018; Di et al., 2020). These results suggested that circRNAs could have important biological functions in the development and environmental information processing of *H. contortus*.

Numerous researches have indicated that circRNAs containing miRNA binding sites act as miRNA decoys or sponges to competitively suppress miRNA activity on their targets (Hansen et al., 2013; Memczak et al., 2013). In the present study, a big number of differentially expressed circRNAs contained one or more potential miRNA binding sites, suggesting that these circRNAs may function as miRNA sponges in *H. contortus*. In *C. elegans*, cel-let-7 was reported to regulate several transcription factors during the developmental transition from larvae to adult worms (Grosshans et al., 2005). Based on the homologous relationship between hco-miR-5991 and cel-let-7 (Winter et al., 2012), and our analytic result that hco-miR-5991 could interact with 167 circRNAs, we speculate that some circRNAs might participate in *H. contortus* larval development *via* the let-7 pathway. In addition, the up-regulation of *C. elegans* miRNA lin-4 reduced the activity of its target, the transcription factor lin-14, which then significantly extended the lifespan of this nematode (Boehm and Slack, 2005). The lin-4-lin-14 pair conferred life span extension through the insulin/insulin-like growth factor-1 pathway (Boehm and Slack, 2005). The mature sequence of cel-lin-4 has high homology with hco-lin-4, which was predicted to be bound by 36 circRNAs, suggesting that some circRNAs could also affect nematode developmental processes through this pathway in *H. contortus*. Also in this parasite, a previous study reported that hco-miR-228 and hco-miR-235 were enriched in L3 (Marks et al., 2019) and our analysis found that hco-miR-228 and hco-miR-235 combined with 78 and 55 circRNAs, respectively, suggesting that some circRNAs may be involved in L3’s development.

Except for being involved in the developmental regulation of nematodes, miRNAs could play roles in drug resistance. The *C. elegans* cel-miR-1 negatively regulated the expression of two nicotinic acetylcholine receptor (nAChR) subunits, UNC-29 and UNC-63, which are targets of levamisole (Simon et al., 2008). Intriguingly, the expression levels of both subunits were increased in *C. elegans* miR-1 mutant strains, whereas the muscle sensitivity to acetylcholine and levamisole was decreased. As hco-miR-1 was homologous to cel-miR-1 and could bind to 19 circRNAs, we surmise that circRNAs may also play functions in drug resistance in *H. contortus*.

Although whether circRNAs can act as miRNA sponges has not been experimentally verified in *H. contortus*, there should be a high probability that this mechanism exists in nematodes, like miRNA sponges in animals that regulate gene expression at the epigenetic level (Luo et al., 2021; Zhou et al., 2021). Certainly, the potential interplay between circRNA and miRNA in parasitic nematodes needs to be further studied in defined biological validation experiments in the future (Li et al., 2018b). Due to the lack of transgenic methods and the restriction of parasitic nematode culture conditions *in vitro*, cross-linking immunoprecipitation (CLIP) approaches can be applied to recognize circRNA-miRNA interactions using the antibody to the Argonaute (AGO) protein. Certainly, functions of more and more circRNAs will be elucidated in the future with the advances of new technologies.

## Conclusion

In summary, the general features and expression profiles of circRNAs at critical stages of *H. contortus* were characterized and bioinformatic analysis revealed that circRNAs could have multiple biological functions during larval developmental processes. Abundant circRNAs contained one or more miRNA potential binding sites, suggesting that they may act as new post-transcriptional regulators involved in the development of *H. contortus*. Therefore, this study provided a basis for elucidating the dynamic regulation of circRNAs in *H. contortus* developmental processes. In addition, parasitic worms had relatively complex gene expression regulation system, so it was of great significance to study non-coding RNAs for revealing the physiological mechanism of parasites and the molecular mechanism of parasitic diseases.

## Data Availability Statement

The data presented in the study are deposited in the NCBI repository, the accession number: PRJNA756739.

## Ethics Statement

Experimental animals used in the project were treated in strict accordance with the Rules for Animal Ethics and Experimentation in the People’s Republic of China. The maintenance of goats was in accordance with protocols approved by The Scientific Ethics Committee of Huazhong Agricultural University (permit HZAUGO-2016-007).

## Author Contributions

MH and CZ contributed to conception and design of the project. YZ, SW, ZW, CW, and FL contributed to animal experiments. CZ and WT performed the experiments and analyzed data. CZ drafted the manuscript, and MH edited and revised the manuscript. All the authors contributed to the article and approved the submitted version.

## Funding

This work was funded by the National Natural Science Foundation of China (NSFC) (Grant no. 31872462 and 321719037) to MH.

## Conflict of Interest

The authors declare that the research was conducted in the absence of any commercial or financial relationships that could be construed as a potential conflict of interest.

## Publisher’s Note

All claims expressed in this article are solely those of the authors and do not necessarily represent those of their affiliated organizations, or those of the publisher, the editors and the reviewers. Any product that may be evaluated in this article, or claim that may be made by its manufacturer, is not guaranteed or endorsed by the publisher.

## Supplementary Material

Supplementary Figure 1 Verification of head-to-tail back-splicing site. Black inverted triangles represented the back-spliced junction loci. Marker: 2000 bp ladder DNA marker.

Supplementary Table 1 Detailed information of convergent and divergent primers used for validating the back-splicing junction sites of circRNAs in *H. contortus*.

Supplementary Table 2 Information on divergent primers for validating the RNA-Seq reliability and the resistance to RNase R digestion of circRNAs in *H. contortus.*

Supplementary Table 3 Summary of sequencing results in *H. contortus* generated from nine libraries.

Supplementary Table 4 Information of all novel circRNAs identified in *H. contortus.*

Supplementary Table 5 The prediction of interaction network for *H. contortus* circRNA-miRNA.

Supplementary Table 6 Information on ten specific binding sites between hco_circ_00015227 and hco-miR-307.

Supplementary Table 7 Primer sequences and product lengths of circRNAs for alternative event.
