# Supplementary figures and images for: Genome-Wide Identification of CircRNAs of Infective Larvae and Adult Worms of Parasitic Nematode, Haemonchus contortus
Source: Front Cell Infect Microbiol. 2021 Nov 22;11:764089. doi: 10.3389/fcimb.2021.764089 (PMC8645938; doi:10.3389/fcimb.2021.764089)

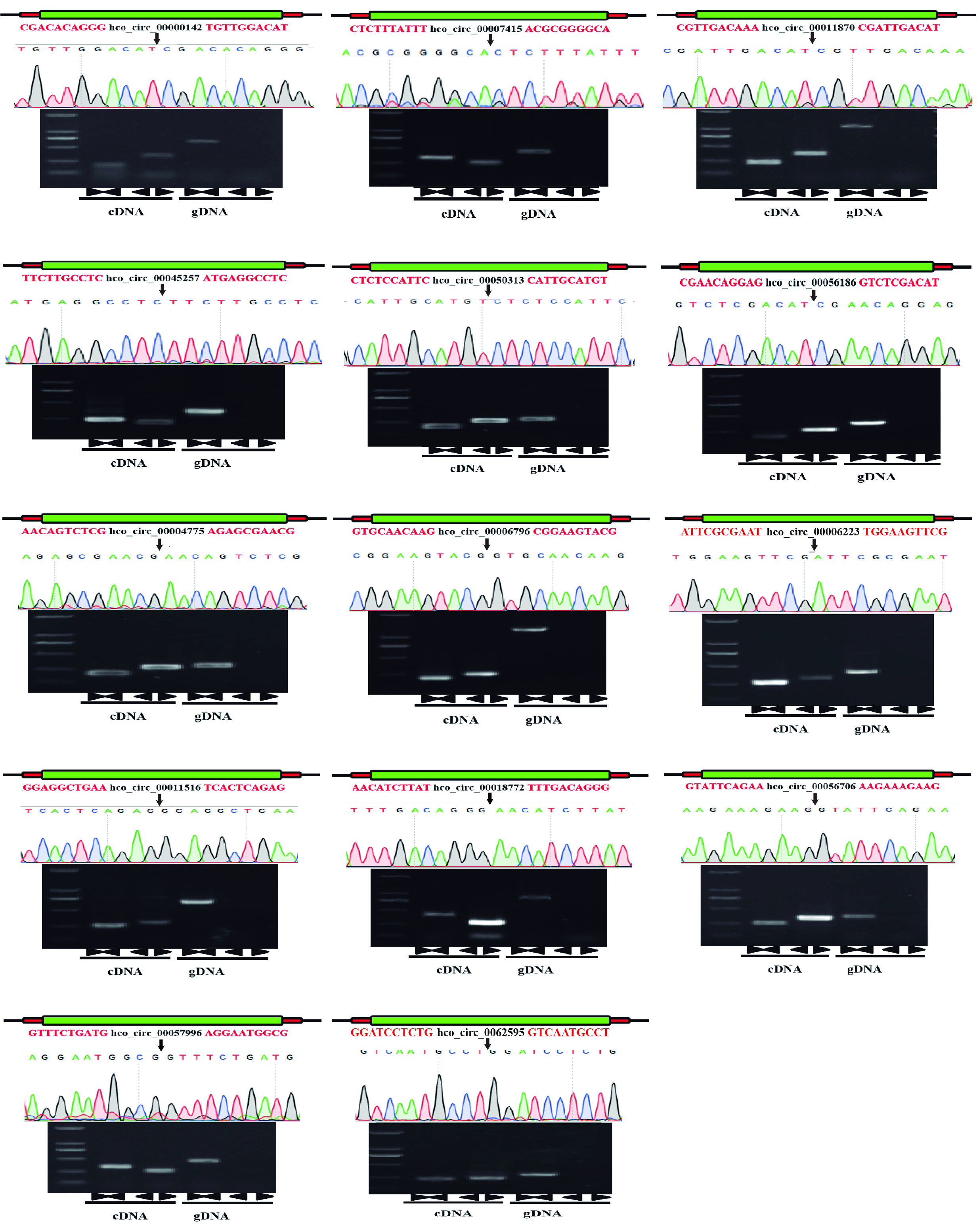

Supplement: Supplementary Figure 1 — Verification of head-to-tail back-splicing site. Black inverted triangles represented the back-spliced junction loci. Marker: 2000 bp ladder DNA marker. [file Image_1.jpeg]
