# Supplementary material for: Genome-Wide Identification of CircRNAs of Infective Larvae and Adult Worms of Parasitic Nematode, Haemonchus contortus
Source: Front Cell Infect Microbiol. 2021 Nov 22;11:764089. doi: 10.3389/fcimb.2021.764089 (PMC8645938; doi:10.3389/fcimb.2021.764089)
Supplement: Supplementary Table 6 — Information on ten specific binding sites between hco_circ_00015227 and hco-miR-307. [file Table_6.docx]

**Table S6. The ten binding site of hco_circ_00015227 and hco-miR-307.**

| circRNA_ID  miRNA_ID | circRNA(top) – miRNA (bottom) paring | site type | circRNA start | circRNA end |
| --- | --- | --- | --- | --- |
| hco_circ_00015227 (5’-3’)  hco-miR-307(3’-5’) | **TGGACGTAGGTAGTGTGGTTGTGG**  **AGCUGAAUGAGUUCCUCCAACACU** | **7mer-m8** | **31** | **54** |
| hco_circ_00015227 (5’-3’)  hco-miR-307(3’-5’) | **TTGTGGTGGTCGGCGAGGTTGTGG**  **AGCUGAAUGAGUUCCUCCAACACU** | **7mer-m8** | **139** | **162** |
| hco_circ_00015227 (5’-3’)  hco-miR-307(3’-5’) | **TTGTAGTCGTCGGCGAGGTTGTGG**  **AGCUGAAUGAGUUCCUCCAACACU** | **7mer-m8** | **67** | **90** |
| hco_circ_00015227 (5’-3’)  hco-miR-307(3’-5’) | **TTGTAGTCGTCGGCGAGGTTGTGG**  **AGCUGAAUGAGUUCCUCCAACACU** | **7mer-m8** | **85** | **108** |
| hco_circ_00015227 (5’-3’)  hco-miR-307(3’-5’) | **TTGTAGTCGTCGGCGAGGTTGTGG**  **AGCUGAAUGAGUUCCUCCAACACU** | **7mer-m8** | **121** | **144** |
| hco_circ_00015227 (5’-3’)  hco-miR-307(3’-5’) | **TTGTAGTCGTCGGCGAGGTTGTGG**  **AGCUGAAUGAGUUCCUCCAACACU** | **7mer-m8** | **157** | **180** |
| hco_circ_00015227 (5’-3’)  hco-miR-307(3’-5’) | **TTGTAGTCGTCGGCGAGGTTGTGG**  **AGCUGAAUGAGUUCCUCCAACACU** | **7mer-m8** | **175** | **198** |
| hco_circ_00015227 (5’-3’)  hco-miR-307(3’-5’) | **TTGTAGTCGTCGGCGAGGTTGTGG**  **AGCUGAAUGAGUUCCUCCAACACU** | **7mer-m8** | **193** | **216** |
| hco_circ_00015227 (5’-3’)  hco-miR-307(3’-5’) | **TTGTAGTCGTCGGCGAGGTTGTGG**  **AGCUGAAUGAGUUCCUCCAACACU** | **7mer-m8** | **103** | **126** |
| hco_circ_00015227 (5’-3’)  hco-miR-307(3’-5’) | **CTAACACGTTTGGCAGTGGTTGTGC**  **AGCUGAAUGAGUU-CCUCCAACACU** | **7mer-m8** | **315** | **339** |
